# Autophagy suppresses cell migration by degrading GEF-H1, a RhoA GEF

**DOI:** 10.18632/oncotarget.8883

**Published:** 2016-04-21

**Authors:** Tatsushi Yoshida, Masatsune Tsujioka, Shinya Honda, Masato Tanaka, Shigeomi Shimizu

**Affiliations:** ^1^ Department of Pathological Cell Biology, Medical Research Institute, Tokyo Medical and Dental University, Bunkyo-ku, Tokyo 113-8510, Japan; ^2^ Laboratory of Immune Regulation, School of Life Sciences, Tokyo University of Pharmacy and Life Sciences, Hachioji, Tokyo 192-0392, Japan; ^3^ Present affiliation: Department of Biochemistry and Molecular Biology, Graduate School of Medical Science, Kyoto Prefectural University of Medicine, Kawaramachi-Hirokoji, Kamigyo-ku, Kyoto 602-8566, Japan

**Keywords:** migration, autophagy, GEF-H1

## Abstract

Cell migration is a process crucial for a variety of biological events, such as morphogenesis and wound healing. Several reports have described the possible regulation of cell migration by autophagy; however, this remains controversial. We here demonstrate that mouse embryonic fibroblasts (MEFs) lacking autophagy protein 5 (Atg5), an essential molecule of autophagy, moved faster than wild-type (WT) MEFs. Similar results were obtained for MEFs lacking Atg7 and unc-51-like kinase 1 (Ulk1), which are molecules required for autophagy. This phenotype was also observed in Atg7-deficient macrophages. WT MEFs moved by mesenchymal-type migration, whereas Atg5 knockout (KO) MEFs moved by amoeba-like migration. This difference was thought to be mediated by the level of RhoA activity, because Atg5 KO MEFs had higher RhoA activity, and treatment with a RhoA inhibitor altered Atg5 KO MEF migration from the amoeba type to the mesenchymal type. Autophagic regulation of RhoA activity was dependent on GEF-H1, a member of the RhoA family of guanine nucleotide exchange factors. In WT MEFs, GEF-H1 directly bound to p62 and was degraded by autophagy, resulting in low RhoA activity. In contrast, the loss of autophagy increased GEF-H1 levels and thereby activated RhoA, which caused cells to move by amoeba-like migration. This amoeba-like migration was cancelled by the silencing of GEF-H1. These results indicate that autophagy plays a role in the regulation of migration by degrading GEF-H1.

## INTRODUCTION

Cell migration is a fundamental process involved in a variety of biological events, such as morphogenesis, wound healing, and immune responses [1–3]. When a cell receives migration signals, dynamic and spatial changes of the cytoskeleton and cell adhesion are induced [4, 5]. The Rho family of small GTPases plays important roles in carrying out these changes by coordinating the cellular responses that regulate actin polymerization, the binding of actin with myosin, the organization of microtubule and intermediate filament networks, and focal adhesion assembly [6–8]. The activity of Rho family members is further regulated by Rho guanine nucleotide exchange factor (Rho GEF), Rho GTPase-activating protein, and Rho guanine nucleotide dissociation inhibitor [9, 10]. Among these, Rho GEFs are considered to play a central role in Rho GTPase regulation [11, 12].

Autophagy is a catabolic process that digests cellular proteins and organelles using lysosomes [13–15]. Autophagy occurs constitutively at low levels and is accelerated by a variety of cellular stressors. In the autophagic process, damaged proteins and damaged organelles are enclosed inside isolation membranes that eventually mature into double-membrane structures called autophagosomes [13–15]. Cellular constituents are digested after the fusion of autophagosomes with lysosomes. The autophagic machinery is driven by more than 30 autophagy-related proteins (Atgs) [16], together with unc51-like kinase 1 (Ulk1), which is a serine/threonine kinase essential for the initiation of autophagy [17, 18]. Autophagy is also regulated by phosphatidylinositol 3-kinase type III, which promotes the invagination of isolation membranes [19]. The subsequent expansion and closure of isolation membranes are mediated by the Atg5–Atg12 pathway and the microtubule-associated protein 1 light chain 3 (LC3) pathway [16]. Although recent studies revealed the existence of an Atg5-independent type of autophagy, as well as functions of Atg5 other than in autophagy [20–22], Atg5 is indispensable for many types of autophagy.

As autophagy is a fundamental cell function, most cellular events are regulated by autophagy, at least to some extent. In most cases, the regulation of these events is performed via the autophagic degradation of specific molecules that are responsible for these events. Therefore, it is important to clarify whether these events are actually regulated by autophagy, and to identify the specific molecules digested by autophagy. There have been several reports suggesting the involvement of autophagy in the regulation of cell migration [23–26]. However, this remains controversial due to conflicting reports; one report suggested that autophagy inhibits cell motility [23–25], whereas another suggested that autophagy enhances cell motility [26]. Therefore, in this study we aimed to elucidate whether and how autophagy regulates cell migration, particularly focusing on molecules degraded by autophagy. Our results indicated the inhibitory regulation of cell motility by autophagy. We also investigated the mechanisms of this regulation, and demonstrated the involvement of GEF-H1, which is a member of the Rho GEFs. GEF-H1 was degraded by autophagy, resulting in a reduction in RhoA activity and cell motility.

## RESULTS

### Lack of Atg5 stimulates cell motility

**Figure 1:**
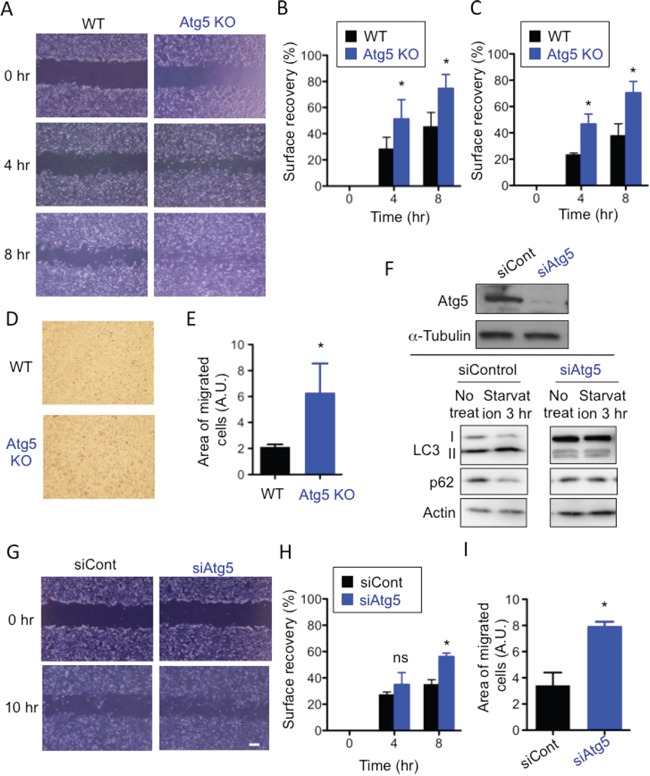
Lack of Atg5 enhances cell migration (A-C) WT MEFs and Atg5 KO MEFs were analyzed using the scratch assay. **A.** Confluent monolayers of WT MEFs and Atg5 KO MEFs were scratched and digital images were acquired at the indicated times. Representative images are shown. **B.** Surface recovery rates were calculated as described in Materials and methods. **C.** The same experiment as in (B) was performed using another set of WT and Atg5 KO MEFs. (D, E) The migratory abilities of WT MEFs and Atg KO MEFs were analyzed using the transwell assay. **D.** Representative images of migrated MEFs stained with Diff-quick. **E.** The area of migrated cells was quantified using Image J software. (F-I) Analysis of Atg5-silenced WT and control WT MEFs. **F.** Successful knockdown of Atg5. (upper panels) Atg5-silenced WT MEFs were verified by Western blotting. α-Tubulin was used as a loading control. (lower panels) MEFs were or were not starved for 3 hr, and cell lysates were subjected to immunoblot analysis using antibodies against LC3, p62, or Actin (as a control). **G, H, I.** Similar experiments as in (A, B, E) were performed using Atg5-silenced WT and control WT MEFs. In (B), (C), (E), (H), and (I), error bars indicate the S.D. (*n* = 5). *p < 0.05.

**Figure 2:**
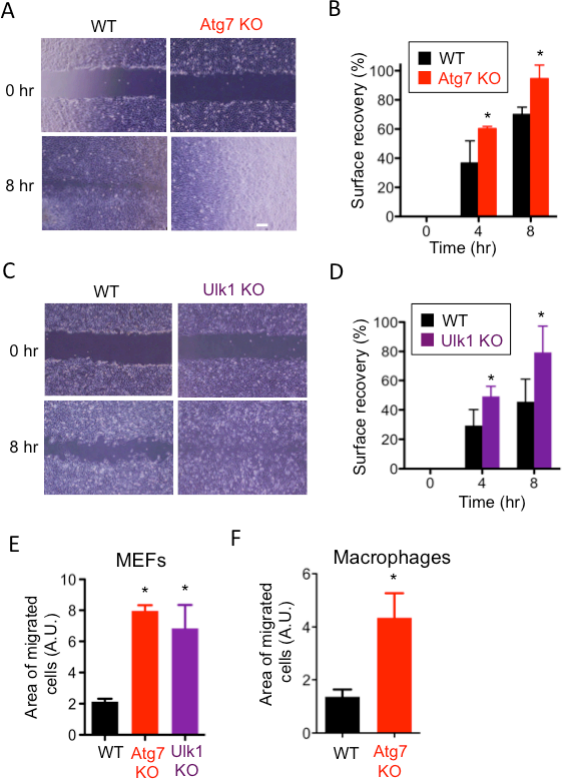
Involvement of Atg7 and Ulk1 in cell migration (A-E) Atg7 KO MEFs and littermate MEFs (A, B, E) or Ulk1 KO MEFs and littermate MEFs (C, D, E) were analyzed by the scratch assay (A-D) or transwell assay (E). **A, C.** Representative digital images of the scratched monolayers acquired at the indicated times. **B, D.** Surface recovery rates were calculated as described in Materials and methods. **E.** The area of migrated cells was quantified using Image J software. **F.** The transwell assay was performed using Atg7-deficient macrophages and WT macrophages. The area of migrated cells was quantified using Image J software. Error bars indicate the S.D. (*n* = 3). *p < 0.05.

While culturing wild-type mouse embryonic fibroblasts (WT MEFs) and Atg5-deficient (Atg5 KO) MEFs, we noticed a faster migration velocity in Atg5 KO MEFs than WT MEFs. This led to our hypothesis that autophagy plays a role in the suppression of cell migration. To test our hypothesis, we performed the scratch migration assay and found that Atg5 KO MEFs moved approximately twice as fast as WT MEFs (Figure 1A, 1B). As the proliferation rate of Atg5 KO MEFs was the same as that of WT MEFs (Suppl. Figure 1), and as we performed this assay in the presence of AraC, which suppresses any effects of cell proliferation, these data simply indicated a difference in moving velocity. Faster movement of Atg5 KO MEFs was also observed when different clones of Atg5 KO MEFs and littermate WT MEFs were compared (Figure 1C, Suppl. Figure 2A). The transwell migration assay also indicated the faster movement of Atg5 KO MEFs than WT MEFs (Figure 1D, 1E). To further confirm the involvement of Atg5 in cell migration, we used Atg5-silenced MEFs. Suppression of autophagy in Atg5-silenced MEFs was verified by the low expression of Atg5, LC3-II and the high expression of p62 after starvation (Figure 1F). As indicated, these MEFs also migrate faster than control MEFs as assessed by the scratch migration assay (Figure 1G, 1H) and the transwell migration assay (Figure 1I). Although the roles of Atg5 other than in autophagy have been reported [22], other autophagy-deficient cells, namely Atg7 KO (Suppl. Figure 2B) and Ulk1 KO (Suppl. Figure 2C, 2D) MEFs also moved faster than littermate WT MEFs (Figure 2A-2E). Importantly, the faster movement by the lack of autophagy was observed not only in MEFs but also in macrophages (Figure 2F). These results indicated that the lack of autophagy stimulates cell motility.

### Atg5 KO MEFs moved by amoeba-like migration

**Figure 3:**
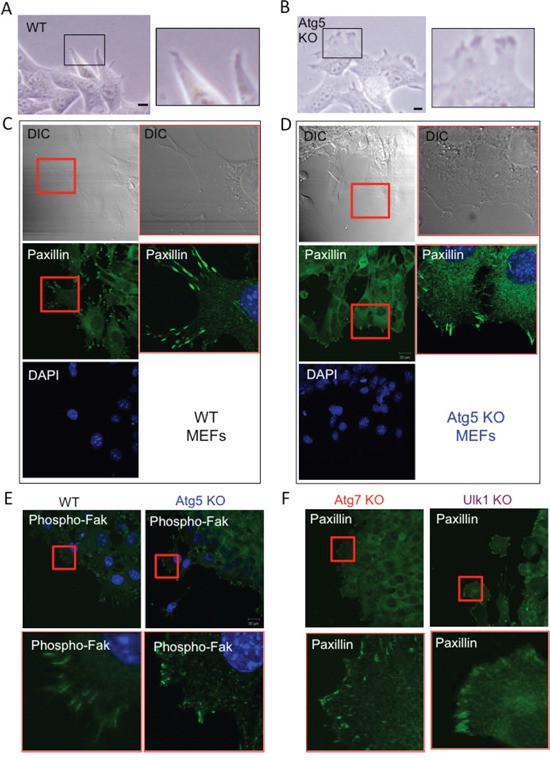
Loss of Atg5 facilitates amoeba-like migration **A, B.** Confluent monolayers of WT MEFs (A) and Atg5 KO MEFs (B) were scratched and the morphologies of their cell edges were observed using a phase-contrast microscope. Magnified images of the rectangular areas are shown on the right. **C, D.** Focal contact assemblies of WT MEFs (C) and Atg5 KO MEFs (D) were examined by paxillin staining. Cells were stained with an anti-paxillin antibody together with DAPI (DNA staining), and observed using a differential interference contrast microscope (DIC) and fluorescence microscope. Magnified images of the rectangular areas are shown on the right. **E.** Focal contact assemblies of WT MEFs and Atg5 KO MEFs were examined by phospho-Fak staining. Magnified images of the square areas are shown in the lower panels. **F.** Focal contact assemblies of Atg7 KO MEFs and Ulk1 KO MEFs were examined by paxillin staining. Magnified images of the square areas are shown in the lower panels.

There are at least two distinct modes of migration; mesenchymal-type migration and amoeba-like migration, and the velocity of amoeba-like migration is faster than that of the mesenchymal type [27–30]. Therefore, we suspected that Atg5 KO MEFs, but not WT MEFs, move by amoeba-like migration. Because cells undergoing mesenchymal-type migration can be distinguished from those moving by amoeba-like migration by examining their leading edge morphology, we examined cells by phase-contrast microscopy. As shown in Figure 3A, WT MEFs had an elongated spindle shape with sharp leading edges, which are features of cells moving by mesenchymal-type migration. In contrast, Atg5 KO MEFs showed rounded edges with small membrane blebs (Figure 3B), which are characteristic features of cells migrating in the amoeboid style. Because the mode of cell migration is reflected by the pattern of focal adhesion assembly, we visualized focal adhesions by staining for paxillin. In WT MEFs, focal adhesions were accumulated and showed rod-shaped staining at the cellular edges, indicative of mesenchymal-type migration (Figure 3C). In contrast, in Atg5 KO MEFs, paxillin was stained broadly (Figure 3D), which is a feature of amoeba-like migration. Despite the different staining patterns of paxillin, its expression level was similar between the two types of MEFs (Suppl. Figure 3). The rod-shaped staining and the broad staining of focal adhesions in WT MEFs and Atg5 KO MEFs, respectively, were confirmed by immunostaining for phosphorylated Fak (Figure 3E). Atg7 KO and Ulk1 KO MEFs showed similar staining patterns of paxillin to Atg5 KO MEFs (Figure 3F). These data indicated that the lack of autophagy facilitates amoeba-like migration and thereby causes a high migration velocity.

### Atg5 KO MEFs show higher RhoA activity than WT MEFs

**Figure 4:**
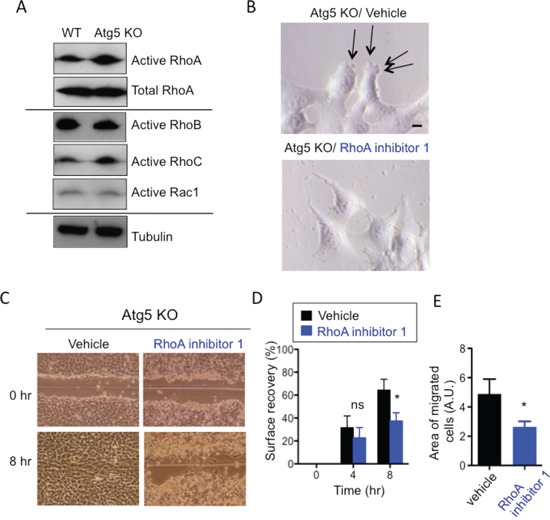
Involvement of RhoA in the amoeba-like migration of Atg5 KO MEFs **A.** Atg5 KO MEFs have higher RhoA activity than WT MEFs. Endogenous active Rho A, B, and C, and Rac1 were measured using the active small GTPases measuring kit, as described in Materials and methods. Total RhoA and tubulin were applied as loading controls. (B-E) Suppression of migratory activity of Atg5 KO MEFs by RhoA inhibitor 1. Confluent monolayers of Atg5 KO MEFs were scratched in the presence or absence of RhoA inhibitor 1 (4 μg/ml). **B.** Morphologies of the cell edges were observed using a phase-contrast microscope. Arrows indicate blebs. **C.** Representative images of control and RhoA inhibitor 1-treated Atg5 KO cells subjected to the scratch assay. **D.** Surface recovery rates calculated from the scratch assay. **E.** Areas of migrated cells calculated from the transwell assay. Error bars indicate the S.D. (*n* = 3). *p* < 0.05. ns indicates not significant vs. value of vehicle.

As cell migration is largely dependent on the Rho/Rho-associated protein kinase (ROCK) signaling pathway [28], we examined the activity of Rho family members by the pull-down assay with Rhotekin-RBD (Rho Binding Domain) beads specifically binding to GTP-bound Rho proteins. Interestingly, the amount of active RhoA in Atg5 KO MEFs was larger than that in WT MEFs, despite them having equivalent total RhoA levels (Figure 4A). We did not observe such differences in the other Rho family GTPases, such as RhoB, RhoC, and Rac-1 (Figure 4A), suggesting that RhoA facilitates the amoeba-like migration of Atg5 KO MEFs. As expected, the addition of Rho inhibitor 1 altered the shape of the leading edges of Atg5 KO MEFs from those of amoeba-like migration to those of mesenchymal-type migration (Figure 4B) and slowed their migration velocity, as confirmed by the scratch migration assay (Figure 4C, 4D) and the transwell migration assay (Figure 4E). In contrast to Rho inhibitor 1, NSC23766, a Rac1 inhibitor, did not alter the cell morphology or cell migration velocity of Atg5 KO MEFs (Suppl. Figure 4). The amount of active RhoA was also larger in Atg7 KO MEFs and Ulk1 KO MEFs than in WT MEFs (Suppl. Figure 5A). Taken together, these results suggest that a deficiency of autophagy activates RhoA, which is crucial for the amoeba-like migration of Atg5 KO MEFs.

### GEF-H1 is degraded by autophagy via its interaction with p62

**Figure 5:**
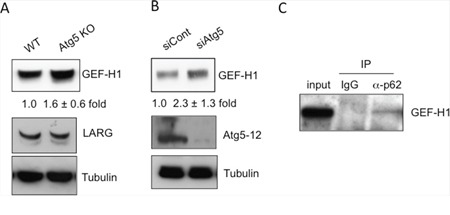
Autophagic degradation of GEF-H1, a Rho GEF (A-C) GEF-H1 levels are increased by the inhibition of autophagy. **A.** Immunoblot analysis of GEF-H1 and LARG in lysates from WT and Atg5 KO MEFs. Tubulin was used as a loading control. The numbers indicate the fold increase in the expression level of GEF-H1 in Atg5 KO MEFs compared to that of WT MEFs. **B.** Immunoblot analysis of GEF-H1 and Atg5-Atg12 complex in lysates from Atg5-silenced MEFs and control MEFs. The numbers indicate the fold increase in expression level of GEF-H1 in siAtg5 MEFs compared to that of siControl MEFs. **C.** Physical interaction between p62 and GEF-H1. WT MEFs were lysed and immunoprecipitated with an anti-p62 antibody or control IgG. Immune complexes and total lysates (10% input) were analyzed by Western blotting using an anti-GEF-H1 antibody.

**Figure 6:**
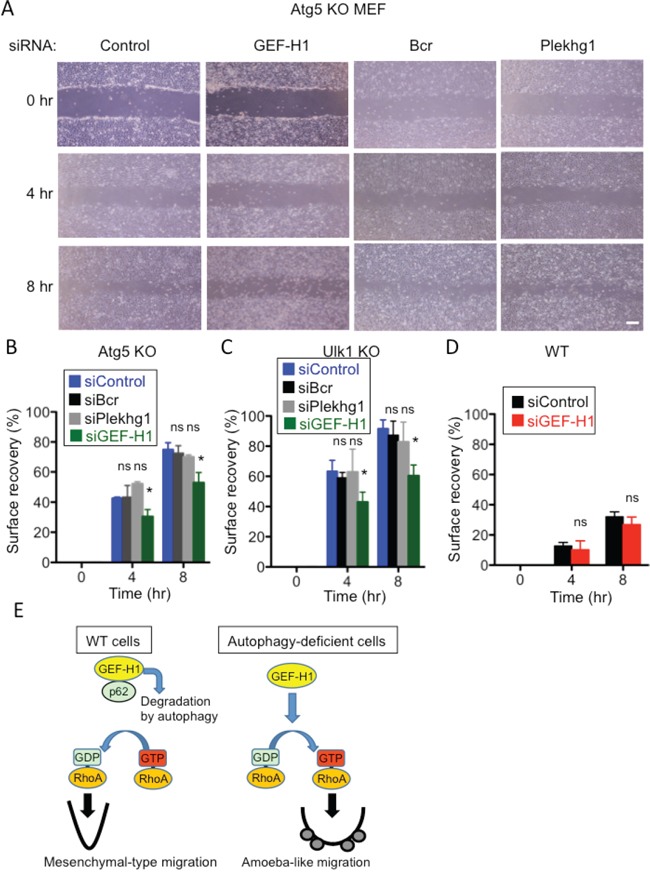
Involvement of GEF-H1 in the migration of Atg5 KO and Ulk1 KO MEFs, but not of WT MEFs **A, B.** Confluent monolayers of Atg5 KO MEFs transfected with the indicated siRNAs were scratched and digital images were acquired at the indicated times. Successful knockdown by each siRNA is shown in Suppl. Figure 6. Representative images are shown in (A) and calculated surface recovery rates are shown in (B). **C, D.** Similar experiments as in (A) were performed using Ulk1 KO MEFs (C) and WT MEFs (D). **E.** Putative scheme of autophagy-dependent regulation of cell motility. In WT cells, autophagy degrades GEF-H1 through its interaction with p62. The reduction of GEF-H1 levels results in the suppression of RhoA activity, which eventually enables cells to undergo mesenchymal-type migration. In autophagy-deficient cells, the increased GEF-H1 levels leads to an increase in RhoA activity, which enables cells to undergo amoeba-like migration. In (B-D), error bars indicate the S.D. (*n* = 5). *p < 0.05. ns indicates not significant vs. value of siControl.

We next investigated the mechanism by which autophagy controls RhoA activity. Rho proteins shuttle between an active (GTP-bound) form and an inactive (GDP-bound) form, and this shuttling is mainly regulated by a GEF that catalyzes the exchange of GDP for GTP [11, 12, 31, 32]. Therefore, we hypothesized that GEFs are involved in the activation of RhoA in Atg5 KO MEFs. We first examined the expression levels of several Rho GEFs in Atg5 KO MEFs and found that the expression level of GEF-H1 was higher in Atg5 KO MEFs than in WT MEFs (Figure 5A). The level of LARG, another Rho GEF, was not increased in Atg5 KO MEFs. An increase in GEF-H1 level was also observed by the transfection of Atg5 siRNA (Figure 5B), indicating that the expression level of GEF-H1 is regulated by autophagy. In most situations of autophagy, p62 binds to autophagy substrates and delivers them to autophagic vacuoles [33, 34]. Therefore, if GEF-H1 is a target substrate of autophagy, it should bind to p62. Immunoprecipitation analysis demonstrated the physical interaction between endogenous GEF-H1 and endogenous p62 (Figure 5C) and this interaction was increased by the induction of starvation-induced autophagy (Suppl. Figure 5B). These results suggested that GEF-H1 is a substrate of autophagic degradation. This led us to hypothesize that an increased level of GEF-H1 induces RhoA activation, thereby resulting in amoeba-like migration in Atg5 KO MEFs. In support of this, the silencing of GEF-H1 decreased the migration velocity of Atg5 KO MEFs (Figure 6A, 6B). Unlike GEF-H1, other Rho GEFs, including Bcr and Plekhg1, did not alter the migration velocity (Figure 6A, 6B). Consistently, the silencing of GEF-H1, but not Bcr or Plekhg1, reduced the migration velocity of Ulk1 KO MEFs (Figure 6C). In contrast, the silencing of GEF-H1 did not alter the migration velocity of WT MEFs, probably because RhoA did not affect mesenchymal-type migration (Figure 6D). The successful knockdown of each gene was confirmed by real-time PCR (Suppl. Figure 6). Taken together, in WT MEFs, autophagy degrades GEF-H1 via its interaction with p62, and the reduction of GEF-H1 results in the suppression of RhoA activity, which eventually enables cells to undergo mesenchymal-type migration.

## DISCUSSION

Several controversial reports have been published regarding the involvement of autophagy in cell motility [23–26]. However, none of these studies showed the detailed molecular mechanisms involved, particularly the specific targets of autophagic degradation. In contrast, in the present study we demonstrated the inhibitory function of autophagy on cell migration and identified the underlying molecular mechanisms. The main findings of this study are as follows: (1) the lack of Atg5, Atg7, and Ulk1 facilitates cell motility in MEFs, and (2) autophagic degradation of GEF-H1 followed by the suppression of RhoA activity is involved in this phenotype. These results indicate that autophagy regulates cell motility via the degradation of GEF-H1.

Cell migration is a multifactorial process in which numerous events occur simultaneously and coordinately [1–3]. The process consists of several steps, including the generation of cell polarity, cell membrane extension, and the formation of focal complexes at the leading edge. Most of these steps are regulated by the Rho family of small GTPases [6, 8]. As Rho GTPases are further regulated by Rho GEF [11, 12, 31, 32], the expression level of Rho GEF is a key factor of cell migration. Therefore, it is reasonable to conclude that the autophagic degradation of GEF-H1 is responsible for the autophagic regulation of cell migration. More than 28 RhoA GEFs have been reported in mammals, and many of them function independently in a cell-type dependent or stimulus-dependent manner [12]. Therefore, although GEF-H1 is the only GEF involved in regulating cell migration in MEFs (as shown here), it is possible that other RhoA GEFs play a role in regulating cell motility in other cells and in response to other stimuli.

Inhibition of the autophagic degradation of GEF-H1 enhances RhoA activity, subsequently leading to the transformation of cells from the mesenchymal type to the amoeboid type. In general, cells that move by mesenchymal-type migration are elongated and spindle-like, and form actin-rich filopodia and lamellipodia at their leading edges [27–30]. The adhesion of these cells to the extracellular matrix (ECM) involves the clustering of integrins mainly in a Rac/Cdc42-dependent manner. In contrast, cells that move by amoeboid-like migration are round with blebbing at the leading edges. Interaction of these cells with the ECM is weak owing to the decreased expression of integrins, and this process is mainly regulated by the Rho/ROCK pathway. Consistent with these facts, Atg5 KO MEFs showed characteristics of cells with amoeboid-like migration, namely, blebbing at the leading edges, weak interaction with the ECM, and regulation by RhoA. Consistent with our findings, other reports described the decreased expression of integrins in Atg5 KO cells [24]. These data altogether indicated that the suppression of autophagy facilitates the transition of cells from the mesenchymal type to the amoeboid type.

Cancer cells are known to have increased migratory activity as they become more malignant, which is mediated by their transformation from the mesenchymal type to the amoeboid type [27, 30]. Amoeboid-like movement enables cancer cells to migrate and metastasize in a protease-independent manner. Therefore, the inhibition of amoeba-like movement by autophagy appears to contribute to the suppression of cancer metastasis. As an oncogenic mutation in Ras was reported to increase the transcriptional expression of GEF-H1 and to activate Rho-dependent amoeba-like movement [35], the activation of autophagy may degrade GEF-H1, suppress amoeba-like movement, and inhibit cancer metastasis.

In conclusion, this study demonstrated that autophagy is crucial for the regulation of cell motility. Furthermore, this function is mediated by the degradation of GEF-H1.

## MATERIALS AND METHODS

### Antibodies and chemicals

Anti-Atg5 and anti-LARG antibodies were purchased from Sigma-Aldrich (St. Louis, MO). Anti-paxillin, anti-p62, anti-actin, and anti-tubulin antibodies were purchased from BD Biosciences (San Jose, CA), MBL (Aichi, Japan), Millipore (Billerica, MA), and Invitrogen (Carlsbad, CA), respectively. Anti-GEF-H1, anti-RhoB, and anti-RhoC antibodies were from Cell Signaling Technologies (Danvers, MA). Anti-RhoA and anti-Rac1 antibodies were from Cytoskeleton Inc. (Denver, CO). Anti-LC3, anti-phosho FAK and anti-GAPDH were purcharsed from NanoTools (Teningen, Germany), Abcam (Cambridge, UK) and Santa Cruz Biotechnology (Santa Cruz, CA), respectively. Bafilomycin A1 was obtained from Sigma-Aldrich. Rho inhibitor 1 was obtained from Cytoskeleton Inc. and Chemdea LLC. (Ridgewood, NJ), respectively. All other chemicals were from Wako Co. (Osaka, Japan).

### Cell culture and DNA transfection

MEFs were prepared from Atg5 KO and Ulk1 KO mouse embryos and their littermates at embryonic day 14.5, and immortalized with SV40 T antigen [36]. Atg7 KO and its control MEFs were a kind gift from Professor Komatsu (Niigata University, Japan). MEFs were cultured in Dulbecco's modified Eagle's medium (DMEM) supplemented with 2 mM L-glutamine, 1 mM sodium pyruvate, 0.1 mM non-essential amino acids, 10 mM Hepes/Na^+^ (pH 7.4), 0.05 mM 2-mercaptoethanol, 100 U/mL penicillin, 100 μg/mL streptomycin, and 10% fetal bovine serum.

For the preparation of bone marrow–derived macrophages, bone marrow cells were obtained by flushing the femurs of 8–10-wk-old C57BL/6 mice or Atg7^F/F^/LysM-cre mice. The cells were treated with Red blood cell lysis buffer (17 mM Tris-HCl [pH 7.5], 144 mM ammonium chloride, and 0.5% fetal calf serum [FCS]) for 1 min at room temperature. Next, the cells were suspended in MEMα medium containing 10% FCS, and were plated at a density of 10^6^ cells/mL in the presence of recombinant mouse macrophage colony-stimulating factor (M-CSF). Cells were harvested on day 3, diluted 1:10 with medium, and cultured for another 3 days. On day 6, the cells were used for the transwell assay [37].

In some experiments, MEFs (1 × 10^6^) were transfected with 10 μg of siRNA using RNAiMax (Thermo Fisher Co. [Waltham, MA]) according to the supplier's protocol. The siRNA sequences used were as follows (the numbers in parentheses indicate nucleotide positions within the respective open reading frame): mouse Atg5, 5′-GAGUCAGCUAUUUGACGUU-3′ (122-140); mouse GEF-H1, 5′-AUCAAUCUUUAUGGACUUCUA-3′ (2143-2163); mouse Bcr, 5′-UCGGUUCACUUUAUUUAUUUA-3′ (5940-5960); mouse Plekhg1, 5′-CUCGUGGUAAAUAGAAAUUUA-3′ (3778-3798); and negative control siRNA.

### Scratch assay

MEFs were grown in poly-L-lysine-coated cell culture dishes. Confluent monolayer cells were scratched with p200 pipette tips or tweezer and incubated at 37°C for various periods of time. AraC (10 μM) was added to the culture medium to inhibit cell growth. Images were acquired at various time points using a microscope. Reference points were set to match the image fields. Straight lines were digitally drawn on the image to mark both borders of the scratch at time 0. Straight lines were again drawn at both cell borders on the image of the same field taken after a certain time. To calculate the recovery rate, the distance between the borders at the two time points on both sides were measured, and the sum of the distances divided by the total distance between the two borders at time 0 was divided by the length of time (Suppl. Figure 7).

### Transwell assay

MEFs or primary macrophages (5 × 10^3^) were added to a cell culture insert (8 μm, Greiner Bio-one). After incubation for 24 hours, the cells were stained using Diff-quick staining kit (Sysmex). Nonmigrated cells on the upper surface were scraped off. Areas of migrated cells on the lower surface were measured using Image J software.

### Paxillin and phospho-Fak staining

Confluent cell monolayers on 12 mm-diameter round cover glasses [Matsunami (Osaka, Japan)] were scratched with p200 pipette tips and incubated at 37°C for 4 hours. The cells were subsequently fixed in 4% paraformaldehyde and permeabilized with 0.1% Triton X-100. The specimens were incubated with mouse anti-paxillin or mouse anti-phospho Fak antibodies, and then with Alexa 488-conjugated goat anti-mouse IgG. Fluorescent images of the specimens were taken by conventional fluorescence microscopy [Olympus Co. (Tokyo, Japan)] or confocal microscopy [Carl Zeiss (Jena, Germany)].

### Analysis of Rho and Rac activities

Rho and Rac activation were quantified using a Rho and Rac activation assay kit (Cytoskeleton, Denver, CO) according to the manufacturer's instructions. Briefly, active Rho proteins in cell lysates were pulled down by Rhotekin-RBD agarose beads, which specifically bind to the GTP-bound forms of Rho proteins, and were analyzed by Western blotting with anti-RhoA, anti-RhoB, and anti-RhoC antibodies. For the Rac activation assay, active Rac proteins were purified by PAK-PBD protein beads and analyzed using an anti-Rac1 antibody.

### Immunoprecipitation and western blotting

The endogenous interaction between p62 and GEF-H1 was assessed by co-immunoprecipitation using an anti-p62 antibody or control IgG. Briefly, MEFs were lysed and immunoprecipitated with an anti-p62 antibody and the amount of co-immunoprecipitated GEF-H1 was estimated by Western blotting.

### Statistical analysis

Results are expressed as the mean ± standard deviation (S.D.). Statistical evaluation was performed using Prism software (GraphPad, La Jolla, CA). Comparisons of two datasets were performed using the unpaired two-tailed Student *t*-test. A *p*-value of less than 0.05 was considered to indicate a statistically significant difference between two groups.

## SUPPLEMENTARY FIGURES


