# Supplementary material for: Autophagy suppresses cell migration by degrading GEF-H1, a RhoA GEF
Source: Oncotarget. 2016 Apr 21;7(23):34420–9. doi: 10.18632/oncotarget.8883 (PMC5085165; doi:10.18632/oncotarget.8883)
Supplement: Supplementary file 1 [file oncotarget-07-34420-s001.pdf]

Autophagy suppresses cell migration by degrading GEF-H1, a RhoA GEF

SUPPLEMENTARY FIGURES

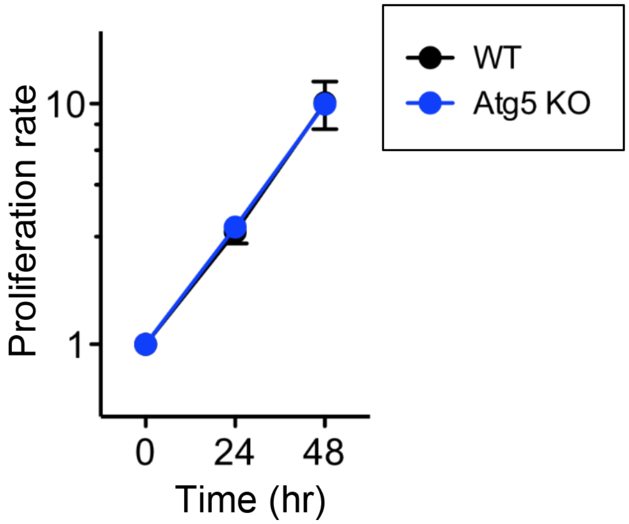

Supplementary Figure S1: Equivalent proliferation rates between WT MEFs and Atg5 KO MEFs. WT MEFs and Atg5 KO MEFs were cultured for 48 hours. At the indicated times, cell numbers were counted.

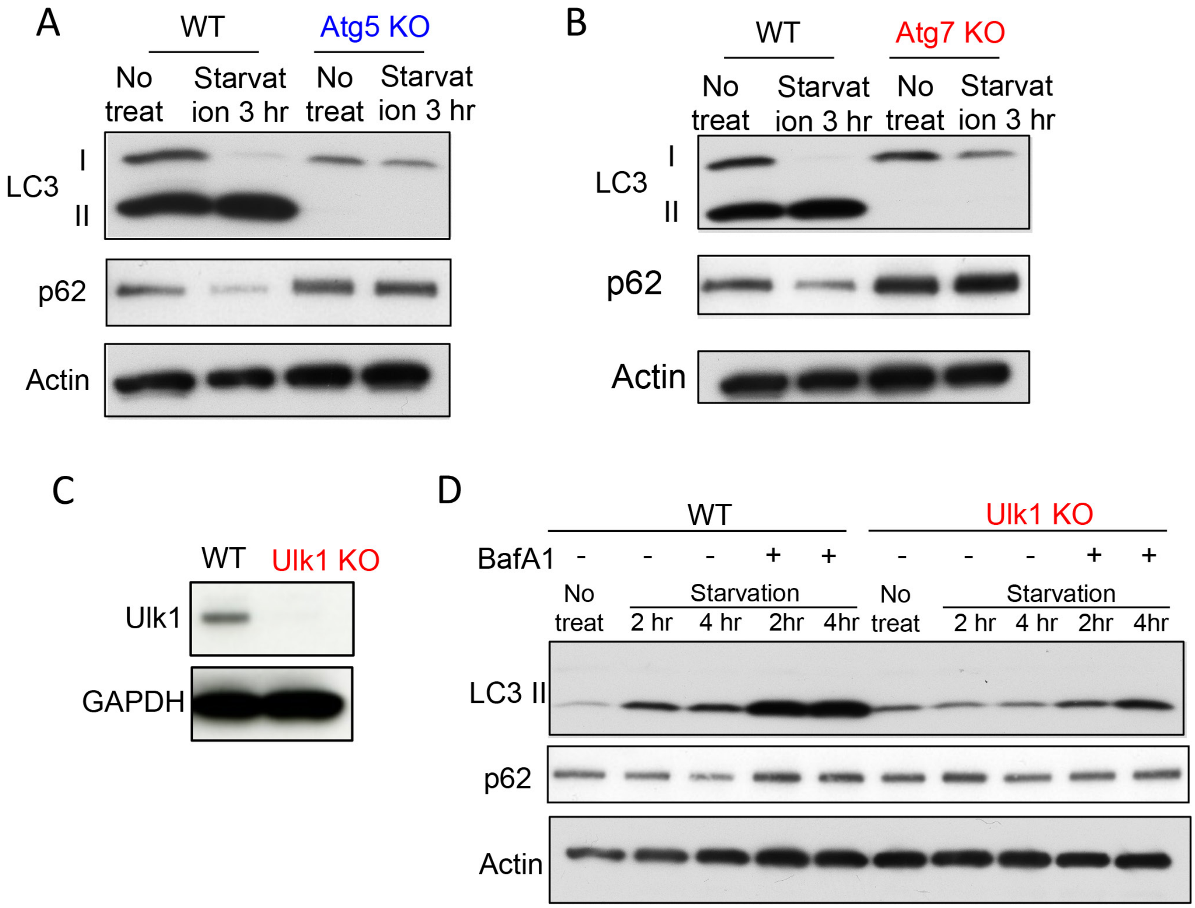

Supplementary Figure S2: Confirmation of Atg5 KO, Atg7 KO, and Ulk1 KO MEFs. A, B. MEFs were or were not starved for 3 hr, and cell lysates were subjected to immunoblot analysis using antibodies against LC3 and p62. Actin was used as a loading control. Absence of LC3-II and accumulation of p62 indicate the lack of autophagy. **C.** Ulk1 KO MEFs were verified by Western blotting. GAPDH was used as a loading control. **D.** WT and Ulk1 KO MEFs were or were not starved for the indicated hours in the presence or absence of Bafilomycin A1 (10 nM). Cells were then harvested, and lysates were subjected to immunoblot analysis using antibodies against LC3 and p62. Actin was used as a loading control. In Ulk1 KO MEFs, autophagy flux is impaired. Autophagy flux is the dynamic process of autophagy, and can be measured by differences in the levels of p62 or LC3-II between the presence and absence of bafilomycin A1. A small difference, which indicates the defect of autophagic flux, was observed in Ulk1 KO MEFs.

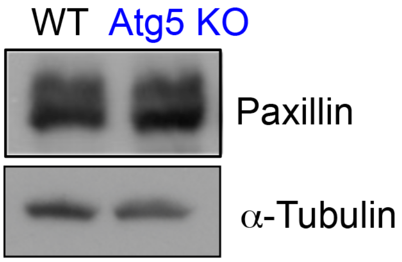

Supplementary Figure S3: Expression levels of paxillin in WT MEFs and Atg5 KO MEFs. Western blot analysis showing equivalent expression levels of paxillin in WT and Atg5 KO MEFs. α-Tubulin is shown as a loading control.

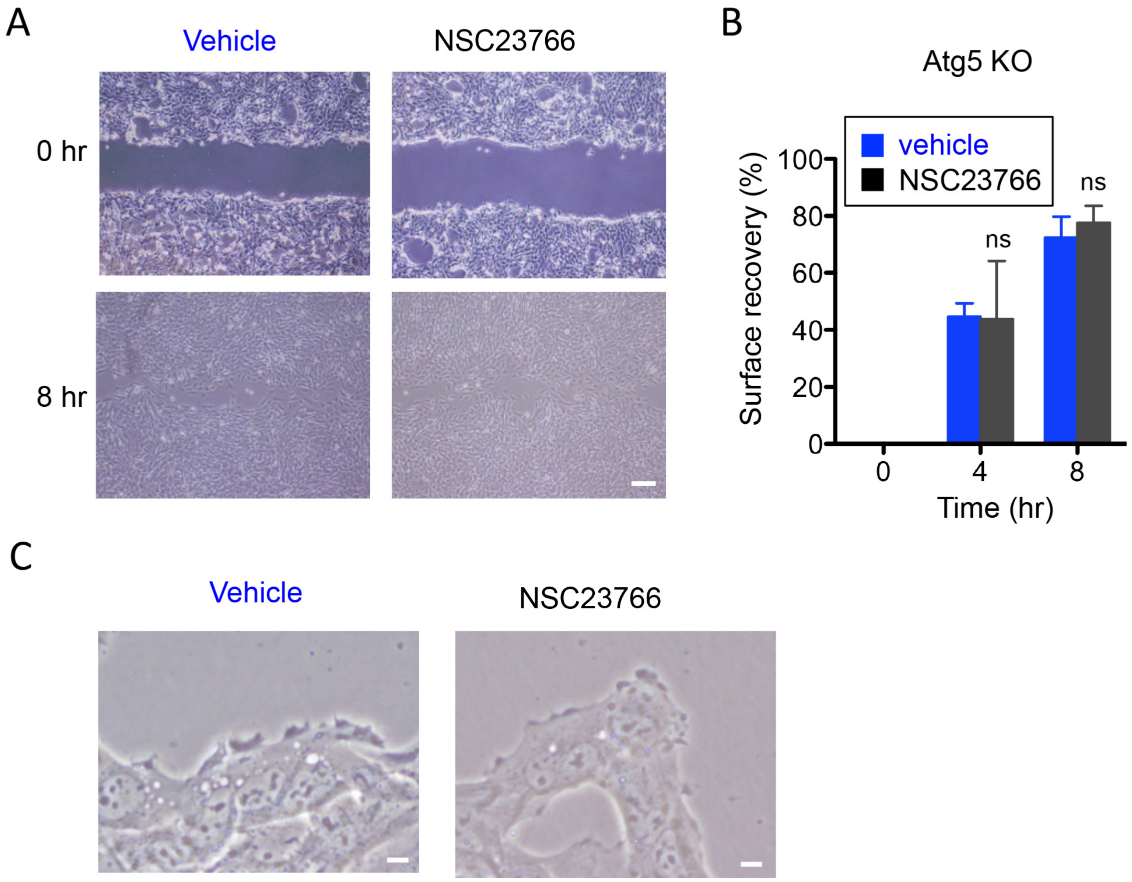

Supplementary Figure S4: A Rac inhibitor does not affect the motility of Atg5 KO MEFs. Confluent monolayer Atg5 KO MEFs, treated with or without 10 µM of NSC23766, a Rac inhibitor, were subjected to the scratch assay. **A, B.** Representative images are shown in (A). Bar = 200 µm. The calculated surface recovery rates are shown in (B). Error bars indicate the SD (n = 3). ns indicates not significant vs. value of vehicle. **C.** Morphologies of the cell edges were observed using a phase-contrast microscope. Bars = 10 µm.

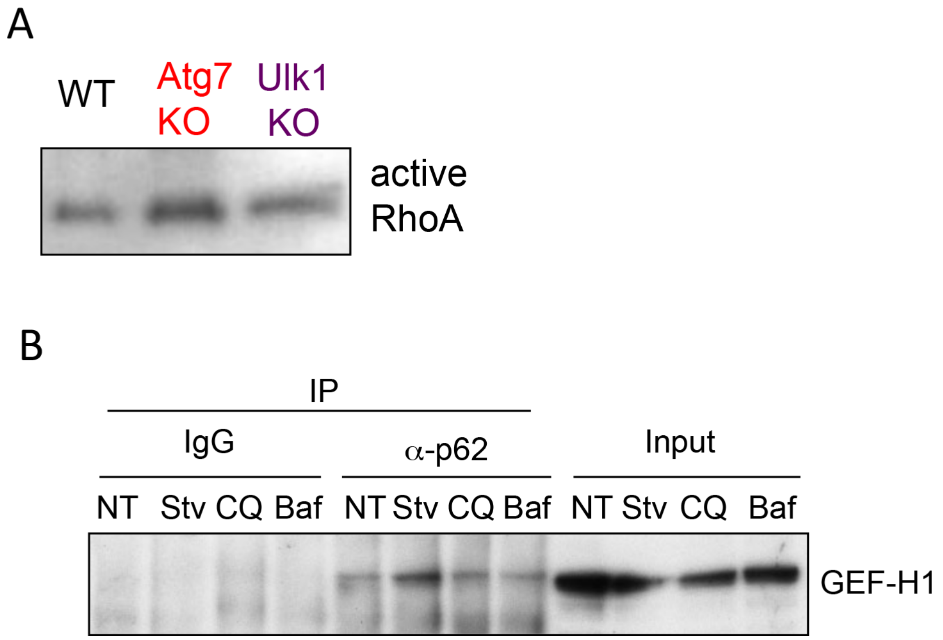

Supplementary Figure S5: Autophagy deficiency enhances RhoA activation through GEF-H1 accumulation. A. Larger amounts of active RhoA in Atg7 KO and Ulk1 KO MEFs than WT MEFs. Endogenous active RhoA was measured using the same method as used in Figure 4A. **B.** Increased interaction between p62 and GEF-H1 by starvation. WT MEFs were starved or treated with chloroquine or bafilomycin A. Then, the physical interaction between p62 and GEF-H1 was examined using the same method as in Figure 5C except that the molecules were crosslinked with dimethyl dithiobispropionimidate (DTBP). Stv; starvation 3 hr, CQ; chloroquine (10 µM), Baf: bafilomycin A1 (10 nM).

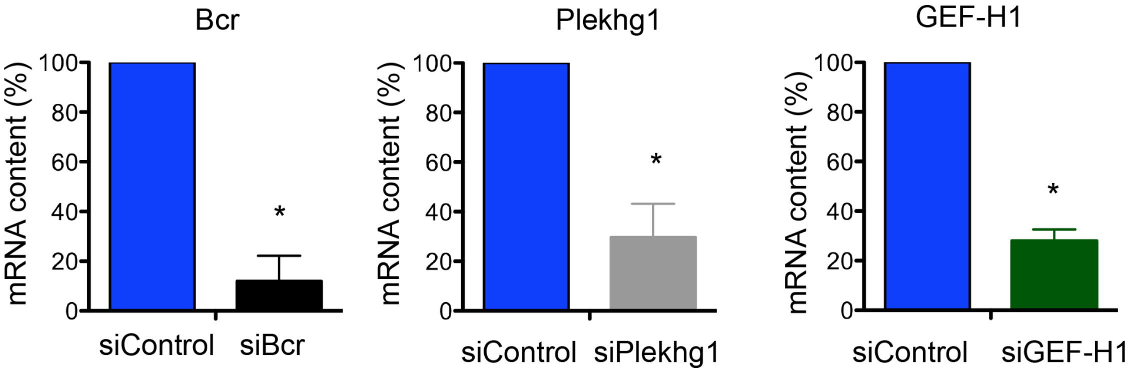

Supplementary Figure S6: Successful knockdown of each Rho GEF by their siRNAs. MEFs were transfected with the indicated siRNAs and the expression level of each gene was assessed by real-time PCR using mRNA. The expression levels of the genes in cells treated with control siRNA were set at 100%. Error bars indicate the SD (n = 3). $*p < 0.05$.

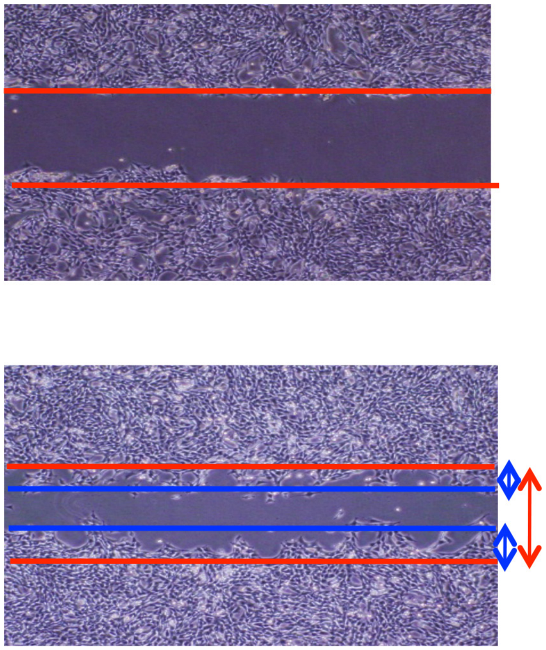

Supplementary Figure S7: Method of the scratch assay. MEFs were grown on poly-L-lysine-coated cell culture dishes. Confluent monolayer cells were scratched with p200 pipette tips and incubated at 37 °C for various periods of time. Cytosine b-D-arabinofuranoside (10 µM) was added to the culture medium to inhibit cell growth. Images were acquired at 0 hr, and at various time points using a microscope. Reference points were set to match the image fields. Straight lines were digitally drawn on the image to mark both borders of the scratch at time 0 (top panel, red lines). Straight lines were again drawn at both cell borders on the image of the same field taken after a certain time (bottom panel, blue lines). To calculate the recovery rate, the distance between the borders at the two time points on both sides were measured (blue double-headed arrows), and the sum of the distances divided by the total distance between the two borders at time 0 (red double-headed arrow) was divided by the length of time.
